# Idiopathic multitudinous fundic gland polyposis: A new disease and a management dilemma

**DOI:** 10.1002/jgh3.12496

**Published:** 2021-02-04

**Authors:** Shiu Kum Lam, George K K Lau

**Affiliations:** ^1^ The Humanity & Health GI & Liver Centre Hong Kong China; ^2^ The University of Hong Kong, Hong Kong Hong Kong China; ^3^ The Humanity & Health Medical Centre Hong Kong China; ^4^ The Liver Diseases & Transplant Centre The Fifth Medical Centre of Chinese PLA General Hospital Beijing China; ^5^ Steering Committee of the Asian Pacific Association for the Study of the Liver Tokyo Japan; ^6^ The Asian Pacific Digestive Week Federation Kuala Lumpur Malaysia

**Keywords:** fundic gland, idiopathic, management, polyps

## Abstract

Two patients with idiopathic multitudinous fundic gland polyposis, a hitherto undescribed condition, were reported. They presented incidentally with a multitude of fundic gland polyps, 52 and 147, without a family history of polyposis, and these polyps were not attributable to the chronic use of proton pump inhibitors. All polyps were removed by hot‐biopsy polypectomy, and each was individually subjected to pathological examination, which showed no evidence of dysplasia. When confronted with gastric polyps of clinically undetermined origin, endoscopists would, to exclude dysplasia, usually resect all if they are few and sample some and survey the others periodically if they are numerous. The condition reported presents a management dilemma: Because the number of the polyps is such that they are manageable by total polypectomy, should this be carried out, despite the labor intensiveness involved, to exclude dysplasia, and are the polyps a variant of syndromic polyposis and therefore carry a malignant potential and inform the need for periodic surveillance and to investigate the patient's kindred? The frequency of this condition and whether it is truly not associated with dysplasia require further studies.

## Graphical abstract

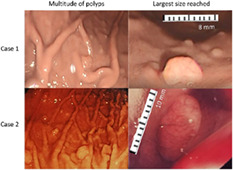

Fundic gland polyps are either sporadic with usually < 10 polyps or syndromic with innumerable polyps. A new condition with 50‐150 fundic gland polyps, with negative family history and not attributable to chronic use of proton pump inhibitors, would present endoscopists a dilemma: whether or not to resect them in total, a labour‐intensive undertaking, to exclude any underlying dysplasia.

## Introduction

Fundic gland polyps are commonly encountered on gastroscopy. They are either sporadic or syndromic in origin.^1^ Sporadic fundic gland polyps, including those associated with the prolonged use of proton pump inhibitors (PPIs) [although the association remains controversial^2^], are mostly fewer than 10 in number,^3^, ^4^, ^5^, ^6^ and a practical approach is to remove all the polyps for pathological examination to exclude dysplasia. In syndromic fundic gland polyposis, including familial adenomatous polyposis, *MUTYH* (MYH) gene*‐associated* polyposis, gastric adenocarcinoma and proximal polyposis of the stomach (GAPPS), and autosomal dominant familial gastric polyposis,^1^ polyps are invariably numerous^7^ and carry a malignant potential in the form of dysplasia in 50–100%.^8^, ^9^ They are usually sampled, and the suspicious ones, such as those ≥10 mm, are resected for pathological examination; the polyps are subsequently surveyed at regular intervals to detect early malignant change.^10^, ^11^

We describe two patients with a multitude of fundic gland polyps of clinically undetermined origin. Because the number of polyps, 52 and 147, were neither few nor numerous, whether these polyps should be removed in total or sampled and periodically surveyed poses a clinical dilemma, with the aim in either way being to detect dysplasia.

## Case Reports

### *Case 1*

**Figure 1:**
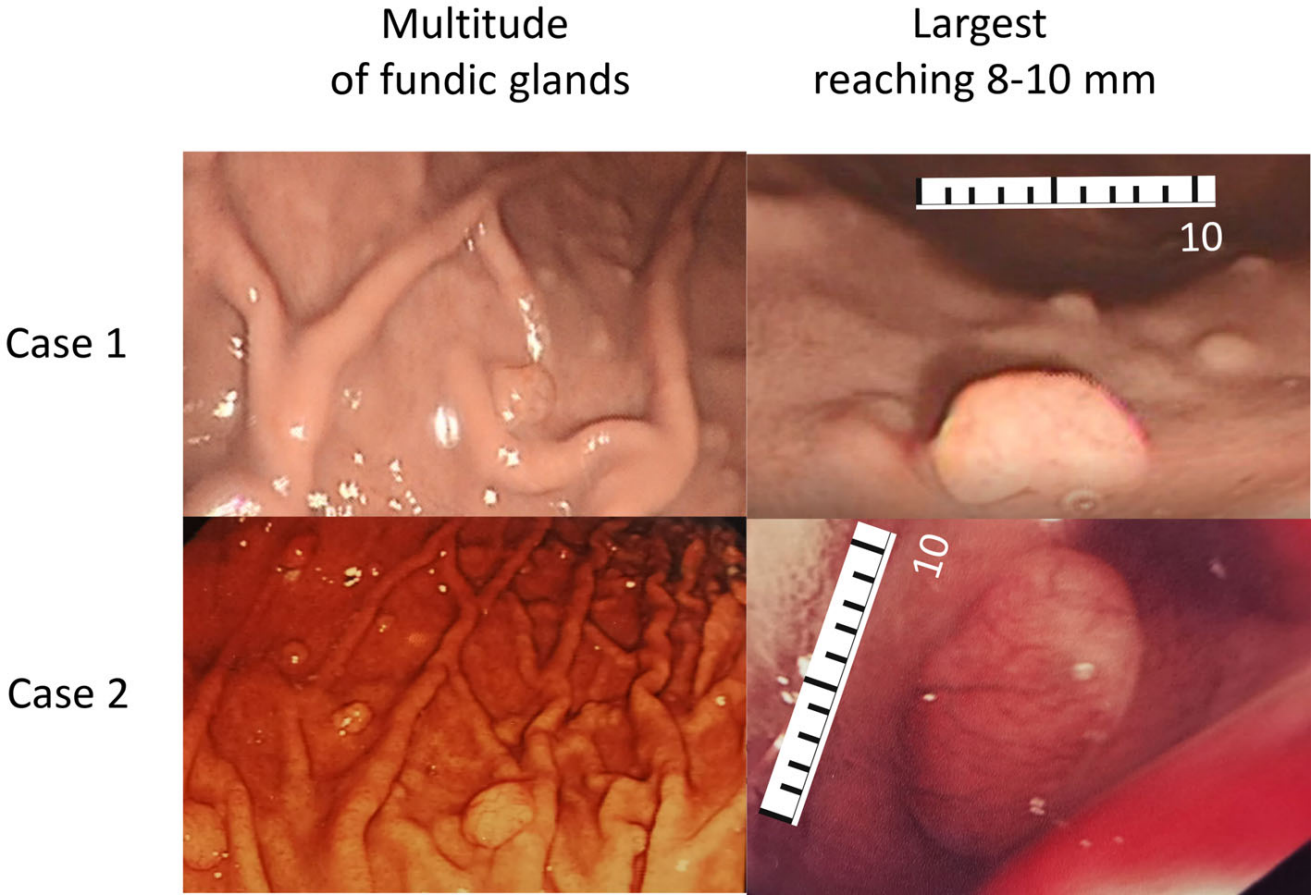
Idiopathic multitudinous fundic gland polyposis. Multitude of polyps in the body of stomach; *n* = 52 in case 1, *n* = 147 in case 2; some polyps reached 8–10 mm.

A 73‐year‐old asymptomatic male with colonic adenoma and polypectomy 3 years earlier underwent colonoscopy and gastroscopy. Colonoscopy revealed three colonic adenomas with mild dysplasia. Gastroscopy revealed a multitude of gastric body polyps 2–8 mm in size (Figure 1, case 1). A total of 52 polyps were completely removed by electrocoagulation using a pair of hot‐biopsy forceps (Olympus FD‐1U‐1, 2.8 mm) in two gastroscopy sessions (approximately 1 h each) 4 weeks apart. The hot‐biopsy approach was used because this would ablate any residual polyp^12^ and reduce the chance of future recurrence at the site. Pathological examination of each individual polyp showed fundic gland polyp without dysplasia. *Helicobacter pylori* was negative by rapid urease test and histology. Endoscopic procedures were carried out under monitored anesthetic care (MAC). The patient did not have a history of taking PPIs; his elder sister had colonic adenoma, and family history of gastric polyposis was negative.

### *Case 2*

A 56‐year‐old female, whose first gastroscopy for heartburn returned a diagnosis of nonerosive gastroesophageal reflux disease and showed no evidence of gastric polyposis, received another gastroscopy 2 years later after 6 months of treatment with a PPI, rabeprazole, 20 mg twice daily for a total of 6 months (three 8‐week prescriptions, 1–3 months apart). This procedure revealed eight polyps in the gastric body. Each polyp was completely removed by electrocoagulation using a pair of hot‐biopsy forceps (same make as Case 1), and pathological examination of each individual polyp showed fundic gland polyp without dysplasia. These polyps were not attributable to PPI use because it is well established that it takes at least 12 months of PPI use for polyps to develop.^3^, ^13^ Two sigmoid hyperplastic polyps were likewise removed at colonoscopy performed in the same sitting. The patient returned after 4 years, during which there was no history of taking rabeprazole or other PPIs, and a gastroscopy carried out to assess recurrence of heartburn revealed a multitude of polyps 2–10 mm in size (Figure 1, case 2). A total of 147 polyps were removed using the same method of hot‐biopsy forceps as in case 1 in 6 endoscopy sessions. Because syndromic polyposis was suspected, each polyp was individually examined pathologically to exclude dysplasia. All polyps were reported to be fundic gland polyps without dysplasia. Endoscopic procedures were carried out under MAC. *H. pylori* was negative by rapid urease test and histology. Family history of gastric or colonic polyps was negative.

## Discussion

Both patients had a multitude of fundic gland polyps, one with 52 and the other with 147. In both cases, the polyps were not attributable to PPI use, and family history was negative for polyposis. This idiopathic multitudinous fundic gland polyposis has hitherto not been described.

Because a syndromic origin was initially suspected, because there are no reliable distinguishing features of dysplasia at endoscopy,^14^ and because the number of polyps was estimated to be manageable by total resection, the polyps in the two patients were removed completely to exclude any possible dysplasia. In both cases, each individual polyp was subjected to pathological examination, and dysplasia was not detected in any.

As family history may not be elicitable in familial syndromic fundic gland polyposis because manifestation in the family may not be apparent or traceable, genetic penetration may be variable, and mutation may occur de novo,^15^ it remains possible that the condition described could be a variant of syndromic polyposis. Furthermore, some polyps reached the size of 8–10 mm, a size more often observed in syndromic than in sporadic polyps.^8^ However, the absence of other syndromic elements, such as polyposis coli, duodenal adenomas, osteomas, or retinal pigmentation,^16^ appear to refute this possibility. Somatic APC (adenomatous polyposis coli) gene alteration^12^ was not measured as there was no reason to suspect familial polyposis coli.

Gastric polyps are commonly encountered on endoscopy. There is no reliable endoscopic feature that correlates with the histology of the polyp;^17^ if the polyps are few, they can all be resected and pathologically examined to exclude dysplasia, and when they are numerous as in syndromic polyposis, they are usually sampled and periodically surveyed for dysplasia.^10^, ^11^ When they are neither few nor numerous, without a family history, and not attributable to PPI use, as in our two cases, there is a management dilemma: Should polypectomy be performed on all the polyps, which would be labor intensive; should they be managed as for syndromic polyps by sampling and surveying them at intervals for malignant potential, an approach that runs the risk of gastric cancer developing during the interval;^18^ and should the patient's kindred be informed that the condition could be a variant of syndromic polyps and, consequently, that they need investigations and follow up for possible polyposis?

There are two possible ways to explain why this condition has not been previously reported. First, it is a rare condition, and second, it is a new disease. Until more cases are accumulated and studied, it remains unknown the frequency with which this condition occurs and whether or not it is associated with dysplasia.

## Declaration of conflict of interest

The authors have no conflict of interest or financial relationship relevant to this article.

## Author contributions

**Shiu K Lam**: Research concept, planning and conduction of the study, collecting and interpretation of data, figures, and writing of the manuscript. **George K K Lau**: Same and equal, viz research concept, planning and conduction of the study, collecting and interpretation of data, figures, and writing of the manuscript.
